# Genomic Insights Into Sugar Adaptation in an Extremophile Yeast *Zygosaccharomyces rouxii*

**DOI:** 10.3389/fmicb.2019.03157

**Published:** 2020-02-11

**Authors:** Hong Guo, Yue Qiu, Jianping Wei, Chen Niu, Yuxiang Zhang, Yahong Yuan, Tianli Yue

**Affiliations:** ^1^College of Food Science and Engineering, Northwest University, Xi’an, China; ^2^College of Food Science and Engineering, Northwest A&F University, Yangling, China

**Keywords:** *Z. rouxii*, *S. cerevisiae*, high sugar stress, osmotic resistance, RNA-sequencing

## Abstract

The osmotolerant *Zygosaccharomyces rouxii* is known for its trait to survive in extreme high sugar environments. This ability determines its role in the fermentation process and leads to yeast spoilage in the food industry. However, our knowledge of the gene expression in response to high sugar stress remains limited. Here, we conducted RNA-sequencing (RNA-seq) under different sugar concentrations of the spoilage yeast, *Z. rouxii*, which exhibit extremely high tolerance to sugar stress. The obtained differentially expressed genes (DEGs) are significantly different to that of the *Saccharomyces cerevisiae*, which is sensitive to extreme high sugar stress. Most of the DEGs participated in the “glucan synthesis,” “transmembrane transport,” “ribosome,” etc. In this work, we also demonstrated that the gene *ZYRO0B03476g* (*ZrKAR2*) encoding Kar2p can significantly affect the growth of *Z. rouxii* under high sugar stress. In addition, we combined with a previous study on the genome sequence of *Z. rouxii*, indicating that several gene families contain significantly more gene copies in the *Z. rouxii* lineage, which involved in tolerance to sugar stress. Our results provide a gene insight for understanding the high sugar tolerance trait, which may impact food and biotechnological industries and improve the osmotolerance in other organisms.

## Introduction

Osmotolerant yeast dominate much of the fermentation process and yeast spoilage in food industries (Watanabe et al., 2004; Dakal et al., 2014; Niu et al., 2016). Most of these yeast species rarely occur on extremely high sugar environments (such as concentrated apple juice, honey, etc.). Among all the osmotolerant yeast, members of the genus *Zygosaccharomyces* are famous not only because of their spoilage ability but also because of their high tolerance to sugar stress (Zuehlke et al., 2013; Alonso et al., 2015; Marvig et al., 2015; Wang et al., 2015; Xiang et al., 2018). *Zygosaccharomyces rouxii* species, which is native to concentrated apple juice, nougat, etc. (Martorell et al., 2007; Wang et al., 2015), is characterized by extraordinary adaptation to sugar stress. Notably, at high sugar stress, it maintains higher growth than other yeast species and can survive at glucose concentrations up to 75% w/v (Dakal et al., 2014). However, 60% w/v extremely high sugar stress was reported to inhibit the growth of *Saccharomyces cerevisiae* (Silva et al., 2005; Dakal et al., 2014). Dakal et al. (2014) have identified *S. cerevisiae* as moderately osmotolerant yeast and *Z. rouxii* as osmotolerant yeast.

Currently, *S. cerevisiae* appears as a cell model to unravel molecular mechanism in response to hyperosmotic stress (Hohmann, 2002; Klipp et al., 2005; De and Al, 2011; Dakal et al., 2014). In *S. cerevisiae*, Hog1 mitogen-activated protein kinase MAPK pathway responds to hyperosmotic stress and ultimately leads to increased synthesis and retention of glycerol (Jiménez-Martí et al., 2011; Dakal et al., 2014; Ene et al., 2015; Mitchell et al., 2015). Several reports have investigated the response of *S. cerevisiae* to mild sugar stress by conducting global or particular transcriptomic analyses. An upregulation of glycerol and trehalose biosynthetic genes was found in *S. cerevisiae* exposed to 20% w/v sugar stress. Erasmus et al. (2003) reported that a particular analysis of the response of wine yeast to 40% w/v sugar stress upregulated the genes of the glycolytic and the pentose phosphate pathway. Jiménez-Martí et al. (2011) also found that the Δ*YHR087W* mutant (*S. cerevisiae*) reduced the expression of heat shock proteins Hsp104 and Hsp78 in response to 20% w/v sugar stress.

Proteomic characterization of *Z. rouxii* response to 60% w/v extreme high sugar stress by our group (Guo et al., 2016) has revealed that most of the differential expressed proteins are involved in carbohydrate and energy metabolism, amino acid metabolism, response to stimulus (mainly heat shock proteins), etc. Among them, Kar2p (belongs to Hsp70 family) is the most prominently upregulated protein, increasing approximately 29-fold. Interestingly, we have unexpectedly discovered that Vaupotic et al. (2008) also reported that in extreme halotolerant black yeast *Hortaea werneckii* and in the adaptation to high amounts of sorbitol (55% w/v), the mitochondria preferentially accumulate Kar2p and Hsp60. However, Pham and Wright (2008) reported that the expression of heat shock proteins remains almost unchanged in *S. cerevisiae* during 30% w/v glucose stress (mild stress). Recently, Liu et al. (2020) found that antioxidant enzymes of *Z. rouxii* under the D-fructose stress were related to the resistance characteristics.

In this study, based on the previous genomics study (Genolévures et al., 2009), we studied the response mechanism of *Z. rouxii* under extreme high glucose stress by physiological response, gene knockout, and global transcriptional response. We further examine gene expression differences following sugar stress treatment in a comparison with *S. cerevisiae*, which is sensitive to extreme high sugar environments. Our study highlighted the genetic bases of sugar tolerance in the *Z. rouxii*.

## Materials and Methods

### Materials

*Zygosaccharomyces rouxii* (BW-WHX-12-54) was isolated from apple juice concentrated by our group (Wang et al., 2015), which was identified by sequencing of the D1/D2 domain of the 26S ribosomal gene and registered at the National Center for Biotechnology Information, United States (GenBank Accession Number KC544459). *S. cerevisiae* (ATCC 38531) was bought from the American Type Culture Collection (ATCC). Phosphate buffer saline (PBS, pH 7.0) and Geneticin (G418) were bought from Sigma-Aldrich (St. Louis, MO, United States). TRIzol was bought from Invitrogen (Carlsbad, United States). M-MuLV reverse transcriptase (RNase H^–^) was bought from Roche (Basel, Switzerland). NEBNext^®^ Ultra^TM^ RNA Library Prep Kit bought from New England Biolabs (NEB, United States). For PCR reactions, Taq DNA polymerase from TaKaRa Biotechnology (Co., Ltd., Dalian, China) was used. Yeast extract, peptone, D-(+)-glucose, and peptide were purchased from local supplier.

### Strain Construction

According to the protein Kar2p detected by Guo et al. (2016), we identified its encoding gene *ZYRO0B03476g* (*ZrKAR2*) in *Z. rouxii*. We designed the primers (*ZrKAR2-F*, *ZrKAR2-R*) to confirm *ZrKAR2*. Then, we designed the interrupt primers (*ZrKAR2-LF*, *ZrKAR2-LR*). All primers are shown in Supplementary Table S1. To delete the *ZrKAR2*, the PUG6 plasmid was used as a template, and *ZrKAR2-LF* and *ZrKAR2-LR* were used as interrupt primers to amplify *KanMX* gene. Then, this *KanMX* PCR product containing a *ZrKAR2* homogeneous arm was obtained and sequenced. The correct PCR products were transferred to *Z. rouxii* according to LiAc methods (Simons et al., 1998). The transformed yeast was applied to a YPD plate containing G418. Positive colonies were confirmed by re-sequencing.

### Strains and Growth Conditions

*Zygosaccharomyces rouxii* BW-WHX-12-54 and *S. cerevisiae* ATCC38531 were grown in YPD (10 g/L yeast extract, 20 g/L peptone, and 20 g/L glucose) broth at 30°C until the culture was in late exponential phase. YPD medium (2% w/v glucose) was used as basic and normal stress medium. YPD containing 40% w/v glucose (40% YPD) and 60% w/v glucose (60% YPD) were used as mild and extreme high sugar stress medium, respectively. For analyses, yeast cells were grown at 30°C. Then, late exponential phase yeast cells (about 2 × 10^9^ CFU/ml) were exposed to 60% YPD, diluted to a density of 2 × 10^7^ CFU/ml for 4 h.

For detecting the growth of *Z. rouxii* and its mutant strain, purified yeast colonies were inoculated to a 200-well plate pre-filled with 250 μl of YPD and 60% YPD medium. Individual growth curves were obtained using an automatic growth curve analyzer (Bioscreen, Finland) at 30°C, the absorbance at 600 nm was measured, and it was automatically detected for 98 h.

### Transmission Electron Microscopy and Atomic Force Microscopy

The transmission electron microscopy (TEM) samples were performed as described previously (Simons et al., 1998), including cutting ultrathin sections by using EM UC7 (Leica, Germany). TEM images were obtained by using a TECNAI G2 SPIRIT TEM (FEI, United States) at an accelerating voltage of 120 kV.

Atomic force microscopy (AFM) images of yeast cells trapped in freshly stripped mica sheet were recorded with a NanoScope V America (Bruker, United States), in contact mode (Pillet et al., 2014), using SiO_4_ cantilevers. Images and cell diameter were analyzed by NanoScope Analysis 1.5.

### Transcriptome Analysis

Late-exponential-phase *Z. rouxii* cells grown in YPD served as the baseline of control (Z_C). Late-exponential-phase *Z. rouxii* (about 2 × 10^9^ CFU) cells were exposed to 60% YPD for 4 h (final density was about 2 × 10^7^ CFU/ml) as stressed-*Z. rouxii* (Z_stress). Late-exponential-phase *S. cerevisiae* cells grown in YPD served as the baseline of control (S_C). Late-exponential-phase *S. cerevisiae* (about 2 × 10^9^ CFU) cells were exposed to 60% YPD for 4 h (final density was about 2 × 10^7^ CFU/ml) as stressed-*S. cerevisiae* (S_stress). *Z. rouxii* cells and *S. cerevisiae* cells were sampled at 4 h after 60% w/v high sugar stress (Supplementary Figure S1). Each sample had three biological replicates. All these 12 samples were harvested and subsequently flash-frozen with liquid nitrogen and stored at −80°C for RNA extraction.

Total RNA was extracted using TRIzol regent (Invitrogen, United States) per the manufacturer’s protocol and treated with RNase-free DNase I. A NanoDrop spectrophotometer (Thermo Scientific, Wilmington, DE, United States), a Qubit Fluorometer 2.0 (Life Technologies, Carlsbad, CA, United States), the agarose gel electrophoresis, and an Agilent 2100 bioanalyzer (Agilent Technologies, Santa Clara, CA, United States) were used to determine concentration, purity, and integrity of RNA samples (Lin et al., 2013; Wang et al., 2018).

The cDNA libraries were constructed according to earlier report (Lai et al., 2017; Wang et al., 2018). A total amount of 1 μg of RNA per sample was used as input material for the RNA sample preparations. Sequencing libraries were generated using NEBNext^®^ Ultra^TM^ RNA Library Prep Kit for Illumina^®^ (NEB, United States) following the manufacturer’s recommendations and index codes were added to attribute sequences to each sample. Briefly, mRNA was purified from total RNA using poly-T oligo-attached magnetic beads. Fragmentation was carried out using divalent cations under elevated temperature in NEB Next First Strand Synthesis Reaction Buffer (5×). First-strand cDNA was synthesized using random hexamer primer and M-MuLV Reverse Transcriptase (RNase H^–^). Second-strand cDNA synthesis was subsequently performed using DNA Polymerase I and RNase H. The cDNA libraries were then sequenced on the Illumina Hiseq2000 platform (San Diego, CA, United States, 2010). FastQC (version 0.11.2) was used for evaluating the quality of sequenced data. The raw reads were generated from control and treatment samples. After filtering the adaptor sequences, the clean reads of *Z. rouxii* and *S. cerevisiae* were mapped onto the reference genome of *Z. rouxii* CBS732 and the reference genome of *S. cerevisiae* S288C by HISAT2 (version 2.0). Gene expression values were quantified by HTSeq v0.6.1. DESeq v1.10.1 was used to determine differentially expressed genes (DEGs) between control and treatment samples. Genes were considered as significant differentially expressed if |log2(fold change)| > 0 and *p-*adj < 0.05. Subsequent enrichment analysis of DEGs by GO enrichment analysis (GOSeq Release2.12) was performed based on a previous report (Wang et al., 2016). The RNA-seq data have been deposited in the National Center for Biotechnology Information (NCBI), with accession code PRJNA437612.

### Validation of RNA-Seq Data by q-PCR

To validate the RNA-seq data, the expression level of five interest genes related to sugar adaptation (2% w/v and 60% w/v) was analyzed by real-time quantitative PCR (q-PCR). The q-PCR experiments were performed according to the method of Guo et al. (2016). Primers were designed using Primer 5, and they are listed in Supplementary Table S1. Total RNA was extracted and purified with Yeast RNAiso kit (TaKaRa Biotechnology Co., Ltd., Dalian, China) following the user protocol. Reverse transcription was performed using PrimeScript^TM^ RT reagent Kit with gDNA Eraser (TaKaRa Biotechnology Co., Ltd., Dalian, China). Real-time quantitative PCR reactions were performed in 96-Well Optical Reaction Plates (Bio-Rad) in trice using the SYBR^®^ Premix Ex Taq^TM^ II (Tli RNaseH Plus) (Takara Biotechnology Co., Ltd., Dalian, China) and analyzed on the iCycler iQ5 2.0 Standard Edition Optical System (Bio-Rad). *ZYRO0F02772g* (*ZrACT1*) was used as the internal control.

## Results

### Overall Description of the Transcriptomic Response to 60% w/v Sugar Stress

To examine the genome-wide responses to sugar stress of this extreme osmotolerant *Z. rouxii*, transcriptome analysis was carried out to assess the specific response at mRNA levels. The details in assembly and annotation information are shown in Table 1. Via RNA-seq, clean reads were obtained. Therein, 95.59 and 94.74% of total clean reads from the Z_C and Z_stress group were aligned to reference sequences (*Z. rouxii* CBS732). 95.31 and 95.53% of total clean reads from S_C and S_stress group were aligned to reference sequences (*S. cerevisiae* S288C). A total of 539 genes in *Z. rouxii* and 3914 genes in *S. cerevisiae* were considered as significant changes in abundance under 60% w/v sugar stress.

**TABLE 1:** Summary of RNA-seq reads in control and treatment groups of *Z. rouxii* and *S. cerevisiae*.

**Parameter**	**Z_C**	**Z_stress**	**S_C**	**S_stress**
Raw reads	36763992	37750001	36529046	34556567
Clean reads	36281618	37260443	35970324	33766773
Total mapped	95.59%	94.74%	95.31%	95.53%
Upregulated genes		247		1959
Downregulated genes		292		1955
Total DEGs		539		3914

**FIGURE 1:**
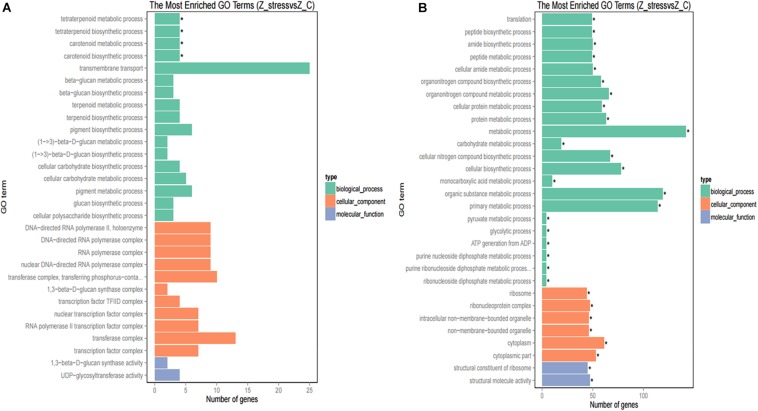
GO functional classification of upregulated DEGs **(A)** and downregulated DEGs **(B)**. The ordinate means GO term, the abscissa means the number of DEGs of each GO term. “*” means significant enrichment.

Furthermore, to better analyze the functions and interactions of the DEGs in *Z. rouxii*, GO enrichment analyses were performed. Figure 1 shows the top 30 enriched functional categories and the bottom 30 enriched functional categories of these DEGs. We found these DEGs of *Z. rouxii* enriched in “glucan biosynthesis” (GO:0051274), “transmembrane transport” (GO:0055085), “ribosome” (GO:0003735), etc. Those DEGs enriched in sensitive pathways will be further analyzed.

**FIGURE 2:**
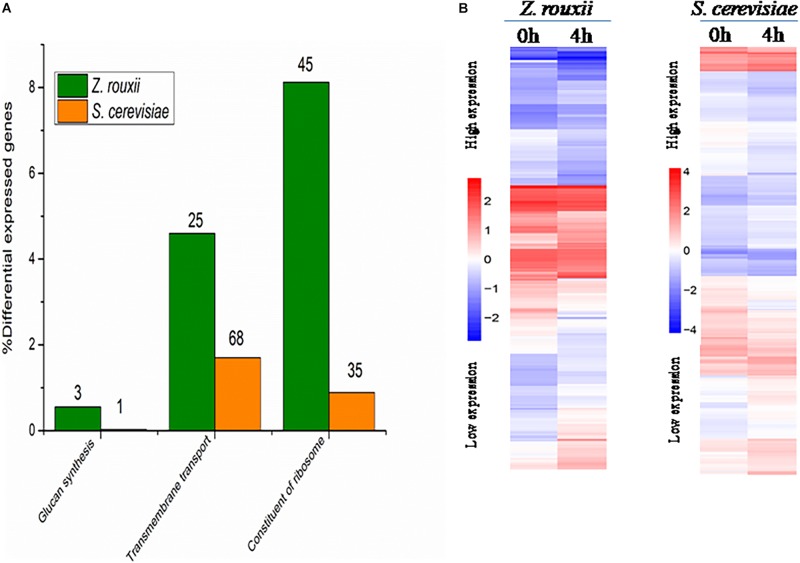
**(A)** DEGs proportions in *Z. rouxii* and *S. cerevisiae*. The number of DEGs of each class is indicated above each bar. **(B)** Expression of the genes identified in *Z. rouxii* and *S. cerevisiae*. The heatmap was generated from hierarchical cluster analysis of genes. The transcript levels were determined by fragments per kilobase of exon per million fragments mapped (FPKM).

Although DEGs of *S. cerevisiae* (3914) were about 7.2 times more than that of *Z. rouxii* (539) (Table 1), *Z. rouxii* had more percent genes enriched in “beta glucan synthesis,” “transmembrane transport,” and “structural constituent of ribosome” involved in sugar stress (Figure 2A). Clustering analysis suggested that many of the genes showed different regulatory patterns in response to sugar between these two species (Figure 2B).

In addition, we combined DEGs of *Z. rouxii* with the gene family analysis of *Z. rouxii* reported by Genolévures et al. (2009). We found that several DEGs we measured belong to *Z. rouxii* specific expansion gene families (Supplementary Table S2). Although these expansion gene families listed in Supplementary Table S2 are come from the genome of *Z. rouxii* CBS 732 (measured by Genolévures et al.), the strain we used in this study is the same species as the one Genolévures et al. (2009) used. Moreover, the reference genome we used is also *Z. rouxii* CBS732 and the alignment rate of the two strains was approximately 95% (Table 1). These DEGs have not been reported in the previous sugar tolerance responses studies (Erasmus et al., 2003; Jiménez-Martí et al., 2011; Dakal et al., 2014). These findings will have important significance for revealing the osmotic stress mechanism of extremophile microorganisms.

Copy number within gene families has been reported to vary greatly between closely related, divergent species (Genolévures et al., 2009; Ma et al., 2013). These gene families related to sugar stress were substantially expanded in *Z. rouxii* compared with other yeast species (Supplementary Table S2). For example, the GL3C0007 gene family, encoding proteins similar to the *FLR1* plasma membrane multidrug transporter, expanded from one member in *Kluyveromyces lactis* genome to four in the *Z. rouxii* genome. Four of these genes occurred as tandem gene array (TGA) in *Z. rouxii* (Genolévures et al., 2009). In this study, we found that *ZYRO0E09966g* in the GL3C0007 gene family was upregulated 2.1-fold in response to extreme high sugar stress. The gene family (GL3C0055) encoding NADPH-dependent oxidoreductase also had more copies (10 copies compared to 0 to 4 for other species) of genes in the *Z. rouxii* genome. Catalase has been reported in affecting D-fructose tolerance of *Z. rouxii* (Liu et al., 2020). The gene family encoding this enzyme was also expanded in the *Z. rouxii*. These findings are similar to the reports of Ma et al. (2013) that several gene families related to high salt stress were substantially expanded in extreme plant desert poplar compared with other plant species.

Furthermore, we verified five interest genes related to sugar adaptation in the RNA-seq data by q-PCR. As shown in Supplementary Figure S2, these results were agreement with the data of transcriptomic analysis. Therefore, it suggested that the RNA-seq data were reliable.

### Cell Wall

**FIGURE 3:**
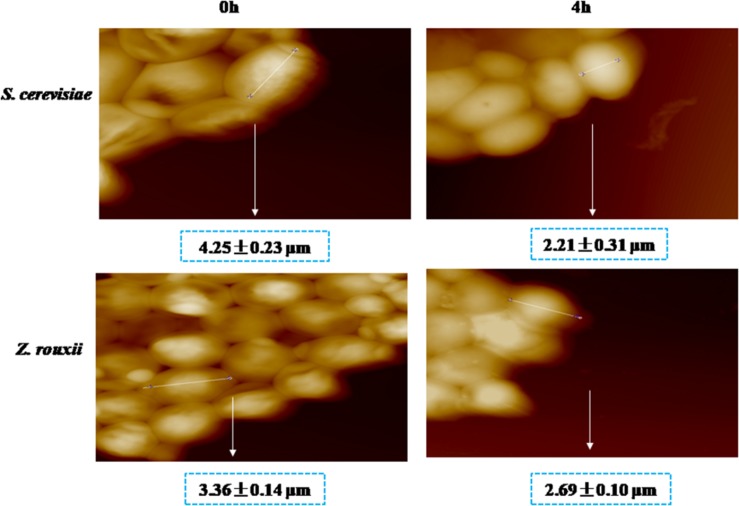
AFM images of *S. cerevisiae* and *Z. rouxii*. Optical images of live native cells.

**FIGURE 4:**
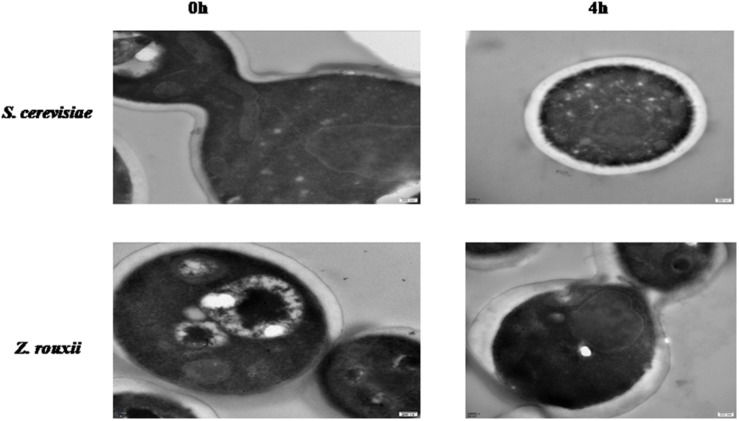
TEM images of cell walls from *Z. rouxii* and *S. cerevisiae*.

Previous reports suggested that the reduction in cell volume during the hyperosmotic stress is accompanied by a thicker β-glucan-chitin layer of the wall (Ene et al., 2015). These phenomena are mediated by cell wall remodeling enzymes (included chitin synthase *CHS1*, β-1,3-glucan synthase *FKS*, cell wall transglycosylases *UTR2*, etc.). In this study, we tested this by comparing the cell size and cell walls of *Z. rouxii* and *S. cerevisiae* cells before sugar addition and after osmo-adaptation. Similar to previous results (Formosa et al., 2013; Ene et al., 2015), there was a significant decrease in cell mean size following sugar stress exposure for both *Z. rouxii* and *S. cerevisiae* cells (Figure 3 and Supplementary Figure S3). Concomitant with the loss in cell volume, the cell wall became thicker (Figure 4).

The upregulated “glucan synthesis” (Figure 1A and Table 2) supported this phenomenon. We found that genes encoding cell wall remodeling enzymes that influence cross-linking in the β-glucan-chitin network exhibited similar and different regulatory patterns in response to 60% w/v sugar stress between *Z. rouxii* and *S. cerevisiae* (Table 2). On the one hand, several genes were both upregulated after 4 h of sugar stress in *Z. rouxii* and high-sugar-sensitive *S. cerevisiae*. For example, *FKS1*, encoding β-1,3-glucan synthase involved in glucan synthesis, affects cell wall integrity (Qiu et al., 2018) and is the target of antifungal agent (Xie et al., 2017). *UTR2*, encoding the putative cell wall transglycosylases, was involved in linking chitin to β-glucan (Brennan et al., 2013; Ene et al., 2015). It has been reported that overexpression of *UTR2* increased the osmotic stress resistance (Ene et al., 2015). In our experiments, *FKS1* and *UTR2* increased similarly in response to sugar stress in *Z. rouxii* and *S. cerevisiae*. Although *UTR2* is not overexpressed in *Z. rouxii*, *Z. rouxii* still has extremely high resistance to osmotic stress. We also found that cell wall remodeling enzymes exhibited the different regulatory patterns in response to 60% w/v sugar stress between *Z. rouxii* and *S. cerevisiae*. For example, the alternative subunit β-1,3-glucan synthase *ZYRO0D06974g* (*FKS3*) and 1,6-glucansynthase encoding gene *ZYRO0G18898g* (*KRE9*) were upregulated after 4 h of sugar stress in *Z. rouxii* but were maintained at control levels in *S. cerevisiae*. In addition, *CHS1*, a gene encoding chitin synthase, is essential for cell wall integrity. The transcript of this gene was upregulated after 4 h of sugar stress in *Z. rouxii*, but was downregulated at control levels in *S. cerevisiae*.

**TABLE 2:** Transcript levels of the genes showing different expression patterns between *Z. rouxii* and *S. cerevisiae* after 60% w/v glucose treatment.

**Function**	***Z. rouxii***	**Fold change**	***S. cerevisiae***	**Fold change**
β-1,3-glucan synthase	*ZYRO0A12518g*	1.6	*FKS1*	1.4
Predicted glucan-chitin cross-linker	*ZYRO0F05280g*	2.6	*UTR2*	2.4
β-1,3-glucan synthase	*ZYRO0D06974g*	1.9	*FKS3*	–
Chitinase	*ZYRO0C10054g*	1.6	*CHS1*	–
β-1,6-glucan synthase	*ZYRO0G18898g*	2.1	*KRE9*	–1.3
Polyamine transporter	*ZYRO0F02090g*	24.8	*TPO1*	–2.2
Ribosomal 40S subunit protein	*ZYRO0G09196g*	–1.8	*RPS13*	2.1
Ribosomal 60S subunit protein	*ZYRO0A03828g*	–1.7	*RPL4B*	2.5

Previous study showed that the degree of cross-linking in the β-glucan-chitin network (a stiffer cell wall) is likely to increase the osmotic resistance of cells and constrains the rate of change in cell size, thereby decreasing compromise cell integrity (Ene et al., 2015). Our results supported this view that the high sugar resistance yeast *Z. rouxii* seems to have a stiffer cell wall (high degree of cross-linking in the β-glucan-chitin network) during the 60% w/v sugar stress and the change amplitude of cell size in *Z. rouxii* was smaller than that of *S. cerevisiae* during the 60% w/v sugar stress.

### Transmembrane Transport

**FIGURE 5:**
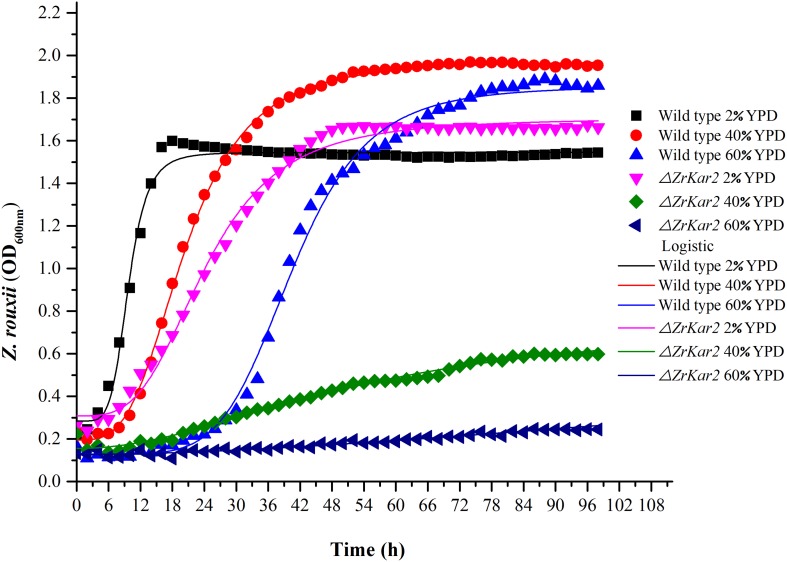
The growth of wild-type strain and mutant strain under 2, 40, and 60% w/v sugar concentrations.

Under 60% w/v sugar stress, 25 genes in Z. *rouxii* involved in transmembrane transport were upregulated, including *ZYRO0E09966g* (*FLR1*), *ZYRO0F02090g* (*TPO1*), *ZYRO0E10054g* (*TPO1*), *ZYRO0G14256g* (*NAB6*), *ZYRO0F17446g* (*GNP1*), *ZYRO0B03784g* (*ORF*), *ZYRO0C08140g* (*FCY2*), *ZYRO0G21252g* (*ZSP1*), *ZYRO0F14652g* (*DTR1*), *ZYRO0C04598* (*THI7*), *ZYRO0F12606g* (*ZRC1*), *ZYRO0B06688g* (*ITR2*), *ZYRO0A00902g* (*PHO84*), *ZYRO0F14630g* (*SSU1*), *ZYRO0A00308g* (*AGP3*), *ZYRO0A04312g* (*ATP16*), *ZYRO0G20614g* (*YHC3*), *ZYRO0E02772g*, *ZYRO0B16896g*, *ZYRO0B03784g*, *ZYRO0G04796g*, *ZYRO0D17732g*, *ZYRO0C06424g*, *ZYRO0E09306g*, and *ZYRO0A11396g*. Among them, polyamine transporter *ZYRO0F02090g* (*TPO1*) was extensively upregulated 24.8-fold after 4 h of sugar stress in *Z. rouxii*, but was downregulated 2.2-fold in *S. cerevisiae*. A previous study indicated that transcript levels of this gene are maintained at control levels in *S. cerevisiae* under 20% w/v and 40% w/v sugar stress (Erasmus et al., 2003; Jiménez-Martí et al., 2011). Krüger et al. (2013) have reported on the function of *TPO1* in response to hydrogen peroxide (H_2_O_2_) stress. They found that *TPO1* controls *S. cerevisiae* cell cycle delay and mediates the induction of antioxidant proteins such as Hsp70 and Hsp90. Furthermore, in overexpressing *TPO1* cells, the induction of Hsps was delayed. Similar to this, in our experiments, *ZYRO0F02090g* (*TPO1*) was upregulated 24.8-fold and *Z. rouxii* had a cell cycle delay (Table 2 and Figure 5). *ZrKAR2*, encoding Kar2p (Hsp70), was downregulated 5.7-fold after 4 h of sugar stress but was increased about 2.9- and 8.2-fold after 8 and 20 h of sugar stress in *Z. rouxii* (Supplementary Figure S4). Then, Kar2p in *Z. rouxii* was significantly increased about 29-fold after about 27 h of 60% w/v sugar concentrations (Guo et al., 2016).

### Sugar Stress Resistance

To further uncover the role of *ZrKAR2*, we deleted this gene. As shown in Figure 5, wild-type strain of *Z. rouxii* grows well at normal sugar concentration (2% w/v), mild high sugar stress (40% w/v), and extremely high sugar stress (60% w/v). Moreover, as the sugar concentration increases, the adaptation time of the wild type to the exponential phase increases (cell cycle delay). However, the growth of Δ*ZrKar2* strain grows well at 2% w/v sugar concentration but was seriously inhibited under both 40% w/v sugar concentration and 60% w/v sugar stress. That is, the deletion of *ZrKar2* did not affect its growth under low sugar conditions but causes the *Z. rouxii* to lose its high sugar tolerance. These results further demonstrated that the gene *ZrKAR2* plays key important roles in the osmotic resistance of *Z. rouxii*. Finally, the logistic equation was applied to fit the growth curve of wild-type and Δ*ZrKar2* mutant strains. Except for the *R*^2^ of the fitting equation of mutant strain during 60% w/v sugar stress (0.95), the *R*^2^ values of other fitting equations are all greater than 0.99, which indicates that the equation can well fit the growth of *Z. rouxii* under different sugar stresses.

In addition, as shown in Figure 5, we speculated that the cell cycle delay in wild-type strains and Δ*ZrKar2* mutant strains was caused by the overexpression of *TPO1* in cells. Due to the limitations of transcriptomics, overexpressed genes often do not necessarily coincide with genes that confer resistance to a particular stressor. Whether overexpression of *TPO1* had the same function as reported in *S. cerevisiae* (Krüger et al., 2013) that controls the cell cycle delay and induces the expression of Kar2p in *Z. rouxii* still requires further verification.

### Ribosome

Most of the genes involved in “ribosome” in the *Z. rouxii* response to 60% w/v sugar concentration were downregulated. This response seems to be consistent with other yeasts under different stresses, for example, *Zygosaccharomyces parabilii* under lactic acid stress (Ortizmerino et al., 2018), *Pichia pastoris* under methanol stress, and *S. cerevisiae* response to silicon encapsulation stress (Fazal et al., 2017). For example, ribosomal protein encoding genes, *RPS13* and *RPL4B*, were downregulated after 4 h (Table 2) of extreme high sugar stress in *Z. rouxii*. Transcript levels of these genes are also strongly downregulated by pH stress in *P. pastoris* (Sauer et al., 2004). It is reported that cell synthesis ribosome requires energy, reducing gene expression associated with ribosome biogenesis and minimizing energy consumption (Fazal et al., 2017). Decreasing “ribosome” seems to be a general stress response in yeast (Fazal et al., 2017).

## Discussion

On the one hand, extreme high sugar tolerance ability makes *Z. rouxii* an increasing threat to high sugary food industries (Dakal et al., 2014; Vermeulen et al., 2015). On the other hand, *Z. rouxii* also played a central role in the production of traditional fermented foods (such as soy sauce) (Watanabe et al., 2013). Extreme yeasts produce and accumulate large amounts of osmo-protective metabolites. Although this feature has been widely exploited in industrial bioprocesses, its molecular mechanism has been poorly investigated (Dakal et al., 2014). In addition, researchers explore how cell adaptation to hyperosmotic stress can help reveal pathological features associated with hyperosmotic stress such as pustular shock, diabetes, etc. (Silva et al., 2005). Combining with existing reports and our group’s research, we considered that *Z. rouxii* is an excellent candidate for analyzing extreme glucose tolerance in cells.

Our protein results (Guo et al., 2016) and gene knockout results (Figure 5) have suggested that *ZrKAR2* protects *Z. rouxii* from the extreme high sugar stress. Simons et al. (1998) demonstrated that cell wall 1,6-β-glucan synthesis in *S. cerevisiae* depends on Kar2p (Hsp70 family). Although this study did not verify the direct relationship between Kar2p and glucan synthesis reported by previous researchers, in our results, both Kar2p and glucan synthesis play an important role in the extreme high glucose tolerance of *Z. rouxii*.

Hsp70 family has been reported to interact with virtually unfolded or misfolded proteins to regulate protein stability and activity (Wang et al., 2004; Hsieh et al., 2013). Hsp90 acts downstream of Hsp70 (Moran Luengo et al., 2018) and its contribution to protein folding is unclear. Moran Luengo et al. (2018) demonstrated that Hsp90 takes a key role in protein folding by breaking the folding barrier caused by Hsp70, empowering protein clients to fold on their own. Hsieh et al. (2013) found that Hsp90 in *Z. rouxii* responded to environmental stress. In our extreme high sugar stress, Hsp90-encoding gene, *ZYRO0E07986g*, was downregulated 5.3-fold. Whether Hsp90 and Kar2p work together to regulate the activity of target protein in *Z. rouxii* during 60% w/v sugar stress still requires further study.

Hao et al. (2018) reported that Hsp70 may modulate stress-activated MAPK signaling by inhibition of p38 (the mammalian homolog of Hog1 from yeast) to protect against heat stress-induced injury in rat small intestine. Glycerol production and retention under hyperosmotic stress are mediated by the Hog1 MAPK signaling cascade, which enhances glycerol synthesis. Recent reports have further confirmed that the Hog1 MAPK pathway is involved in both salt stress and sugar stress (Jiménez-Martí et al., 2011; Guo et al., 2016; Solieri et al., 2016; Wang et al., 2019). Combining recent reports with our results, we found the overlapping subset of salt- and sugar-responsive genes. For example, Iwaki et al. (2001) demonstrated that transcripts of *ZrGCY1* and *ZrGCY2* genes increased in salt-stressed (12% w/v) ATCC42981 cells compared to unstressed ones, suggesting that this salt concentration elicits the Gcy-Dak pathway (Iwaki et al., 2001), which includes the oxidation of glycerol to dihydroxyacetone (DHA). But in our experiments, *ZYRO0F10032g* (*GCY1*) decreased. These results highlighted that yeasts exploit different strategies to adapt in osmotic and salt stress. Solieri et al. (2014) also suggested that the differences in stress response could imply different adaptation mechanisms to sugar stress and salt stress. We speculated that yeast cell downregulated *GCY1* in a high glucose environment in order to retain glycerol.

In addition, we also found the overlapping subset of salt- and sugar-responsive genes related to “structural constituent of ribosome.” We found that in our experiment condition, ribosome protein encoding genes, *ZYRO0G11000g* (*RPL38*) and *ZYRO0A12606g* (*RPL26*), were upregulated in *Z. rouxii* exposed to extreme high sugar stress but were downregulated in *Z. rouxii* under 12% w/v salt stress. These results also indicated that yeast have different strategies in response to sugar and salt stress.

**FIGURE 6:**
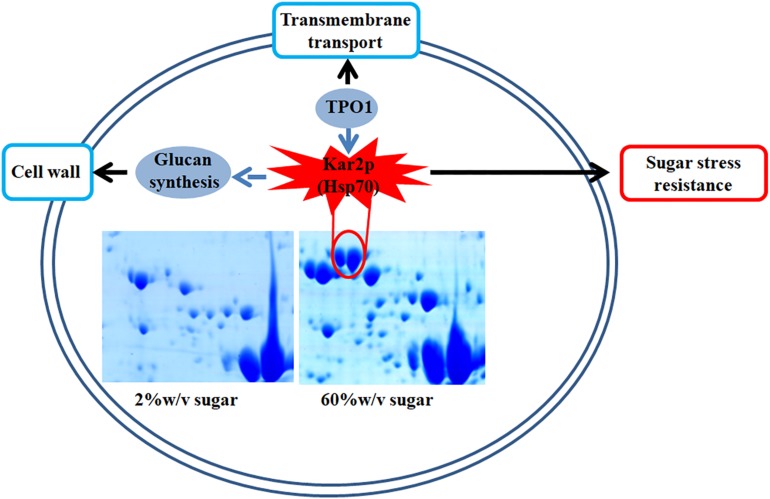
A schematic diagram illustrating the potential relationship of Kar2p (Hsp70 family), glucan synthesis, transmembrane transport, and sugar stress resistance.

In this study, we found that several genes involved in extreme high sugar stress tolerance, such as “transmembrane transport,” were selectively expanded in the *Z. rouxii* genome (Supplementary Table S2). In addition, we demonstrated by gene knockout that *ZrKAR2* encoding Kar2p (Hsp70) significantly affected the growth of *Z. rouxii* at high sugar concentrations. According to previous reports and our results, we found that Kar2p in *Z. rouxii* seems to be associated with “glucan synthesis” and “transmembrane transport” and contributes to sugar stress resistance (Figure 6). Our analyses taken together suggest that *Z. rouxii* may have increased its sugar tolerance through duplication and/or mediates multiple genes involved in transmembrane transport, cell wall remodeling enzymes, ribosome, and Hsp defense response. These findings are important for an improved understanding of yeast adaptation to sugar stress and engineering the osmotolerance in other organisms.

## Data Availability Statement

The datasets generated for this study can be found in the RNA-seq data have been deposited in the National Center for Biotechnology Information (NCBI), with accession code PRJNA437612.

## Author Contributions

HG and TY conceived and designed the experiments. HG performed the experiments. HG, YQ, YY, and CN analyzed the data. HG, JW, and YZ drafted the manuscript. All authors read and approved the final version of the manuscript.

## Conflict of Interest

The authors declare that the research was conducted in the absence of any commercial or financial relationships that could be construed as a potential conflict of interest.
