# Supplementary material for: Genomic Insights Into Sugar Adaptation in an Extremophile Yeast Zygosaccharomyces rouxii
Source: Front Microbiol. 2020 Feb 11;10:3157. doi: 10.3389/fmicb.2019.03157 (PMC7026193; doi:10.3389/fmicb.2019.03157)
Supplement: Supplementary file 1 [file Data_Sheet_1.pdf]

Supplementary Material

Table S1. Primers used in this study.

Primers	5'-3' sequence	Function
ZrKAR2 F	AAGGATGCTGTGGTTACTG	Upstream primer
ZrKAR2 R	GGTTGGTTCGTTGATGATAC	Downstream primer
ZrKAR2-LF	AAGGATGCTGTGGTTACTGCAGCTGAAGCTTCGTACGC	With upstream homologous arm of *ZrKAR2*, amplifying *KanMX* gene
ZrKAR2-LR	GGTTGGTTCGTTGATGATACGCATAGGCCACTAGTGGATCTG	With downstream homologous arm of *ZrKAR2*, amplifying *KanMX* gene
ZYRO0F02090g F	CTGGCGTTGGGTAGAAGGTA	Upstream primer
ZYRO0F02090g R	GCTACCCATCTTTCATCGCC	Downstream primer
ZYRO0E10054g F	ATCGGTTTGGCATGCATCTC	Upstream primer
ZYRO0E10054g R	AACCGAAAGCCACGAGACTA	Downstream primer
ZYRO0B16764 F	TAGGACCCAACTTCAGCCAG	Upstream primer
ZYRO0B16764 R	GTCCTTCTCTGTAGCTCCCC	Downstream primer
ZYRO0A12606g F	GGTGTCAGAGCTTTGCCAAT	Upstream primer
ZYRO0A12606g R	AGCCTTTCTGTCCTTGTCCA	Downstream primer
ZYRO0F02772g F	GCCGTTTTCCCATCCATTGT	Upstream primer
ZYRO0F02772g R	GTGGTGCCAGATCTTTTCCA	Downstream primer

Table S2. Expansion gene families. DEGs of *Z. rouxii* under 60%w/v sugar concentrations were colored red.

Protein family	Copy number	Gene	Short description
GL3C0007	4	*ZYRO0E09966g*	Similar to YBR008C FLR1 Plasma membrane multidrug transporter member of the major facilitator superfamily
(TGA)		*ZYRO0E09988g*	
		ZYRO0E09922g	
		ZYRO0E09900g	
GL3C0447	2	*ZYRO0G08140g*	Similar to SACE YML117W NAB6 Putative RNA-binding protein
(TGA)		*ZYRO0G14256g*	
GL3C0055	10	*ZYRO0F05214g*	Similar to SACE YOL151W GRE2 NADPH dependent methylglyoxal reductase (D- lactaldehyde dehydrogenase)
			Similar to SACE YOL151W GRE2 NADPH dependent methylglyoxal reductase (D- lactaldehyde dehydrogenase)
		ZYRO0G15400g	
			Similar to SACE YOL151W GRE2 NADPH dependent methylglyoxal reductase (D- lactaldehyde dehydrogenase)
		ZYRO0F18656g	Similar to SACE YGL039W and YGL157W
			Similar to SACE YGL039W and YGL157W
			Similar to SACE YGL039W and YGL157W
		ZYRO0G00242g	Similar to SACE YGL039W and YGL157W
		ZYRO0G22506g	Similar to SACE YGL039W and YGL157W
		ZYRO0C18546g	Similar to SACE YGL039W and YGL157W
		ZYRO0A13574g	Similar to SACE YGL039W and YGL157W
		ZYRO0B00330g	
		ZYRO0B16742g	
		ZYRO0E10362g	

GL3C0080	6	*ZYRO0C11902g*	Similar to SACE YHR179W OYE2 Widely conserved NADPH oxidoreductase containing flavin mononucleotide (FMN)
		ZYRO0C11924g	Similar to SACE YHR179W OYE2 Widely conserved NADPH oxidoreductase containing flavin mononucleotide (FMN)
		ZYRO0F12144g	Similar to SACE YHR179W OYE2 Widely conserved NADPH oxidoreductase containing flavin mononucleotide (FMN)
		ZYRO0F13618g	Similar to SACE YHR179W OYE2 Widely conserved NADPH oxidoreductase containing flavin mononucleotide (FMN)
		ZYRO0F16852g	Similar to SACE YHR179W OYE2 Widely conserved NADPH oxidoreductase containing flavin mononucleotide (FMN)
		ZYRO0G15444g	Similar to SACE YHR179W OYE2 Widely conserved NADPH oxidoreductase containing flavin mononucleotide (FMN)
GL3C0186	6	*ZYRO0A01122g*	Similar to SACE YOR388C FDH1 NAD( )-dependent formate dehydrogenase
		ZYRO0A08206g	Similar to SACE YOR388C FDH1 NAD( )-dependent formate dehydrogenase
		ZYRO0D10780g	Similar to SACE YOR388C FDH1 NAD( )-dependent formate dehydrogenase
		ZYRO0F16874g	similar to SACE YNL274C Putative hydroxyisocaproate dehydrogenase
		ZYRO0G18876g	Similar to SACE YOR388C FDH1 NAD( )-dependent formate dehydrogenase
		ZYRO0G19866g	Similar to SACE YOR388C FDH1 NAD( )-dependent formate dehydrogenase

GL3R0232	5	*ZYRO0B16764g*	Similar to SACE YDR256C CTA1 Catalase A breaks down hydrogen peroxide in the peroxisomal matrix
		ZYRO0E10318g	Similar to SACE YDR256C CTA1 Catalase A breaks down hydrogen peroxide in the peroxisomal matrix
		ZYRO0F08426g	Similar to SACE YDR256C CTA1 Catalase A breaks down hydrogen peroxide in the peroxisomal matrix
		ZYRO0C17820g	Similar to SACE YGR088W CTT1 Cytosolic catalase T has a role in protection from oxidative damage by hydrogen peroxide
		ZYRO0G22462g	Similar to SACE YGR088W CTT1 Cytosolic catalase T has a role in protection from oxidative damage by hydrogen peroxide
GL3C0522	4	*ZYRO0A00286g*	Similar to SACE YPL088W Putative aryl alcohol dehydrogenase
		ZYRO0D02816g	Similar to SACE YPL088W Putative aryl alcohol dehydrogenase
		ZYRO0F04840g	Similar to SACE YPL088W Putative aryl alcohol dehydrogenase
		ZYRO0G20504g	Similar to SACE YPL088W Putative aryl alcohol dehydrogenase

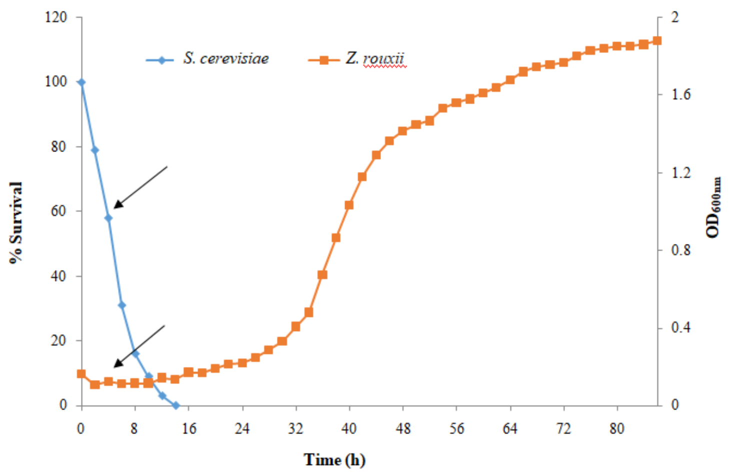

Figure S1 Effect of 60%w/v sugar stress on the *Z. rouxii* and *S. cerevisiae*.

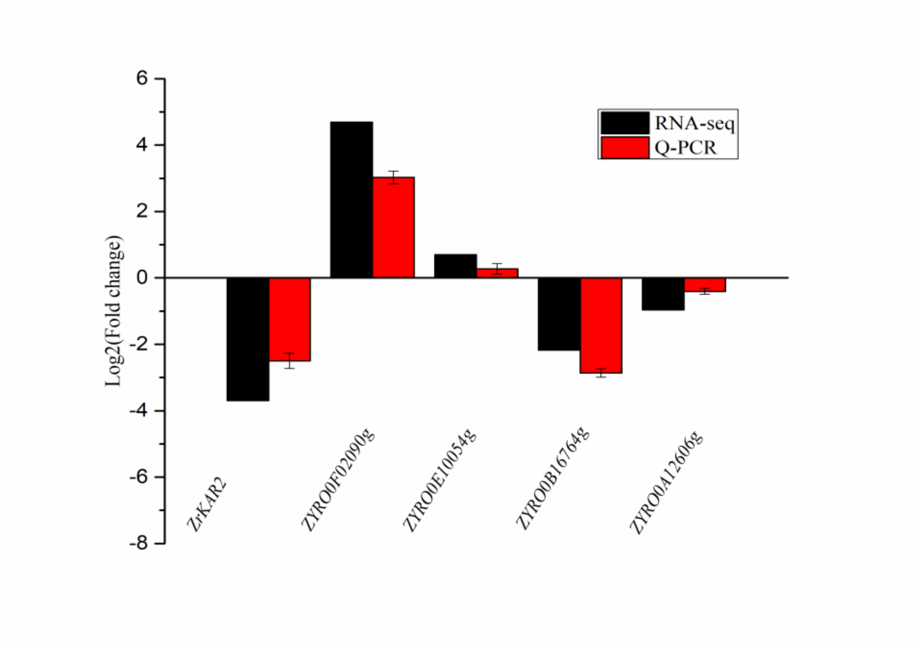

Figure S2 Validation of RNA-seq data was performed by q-PCR.

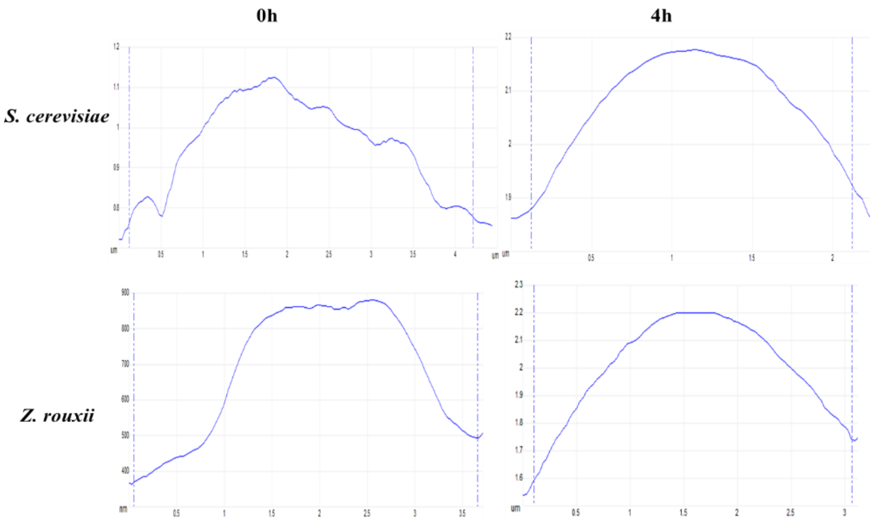

Figure S3 AFM images of *S. cerevisiae* and *Z. rouxii*. Cross sections take along the lines on the height images corresponding to Fig. 4

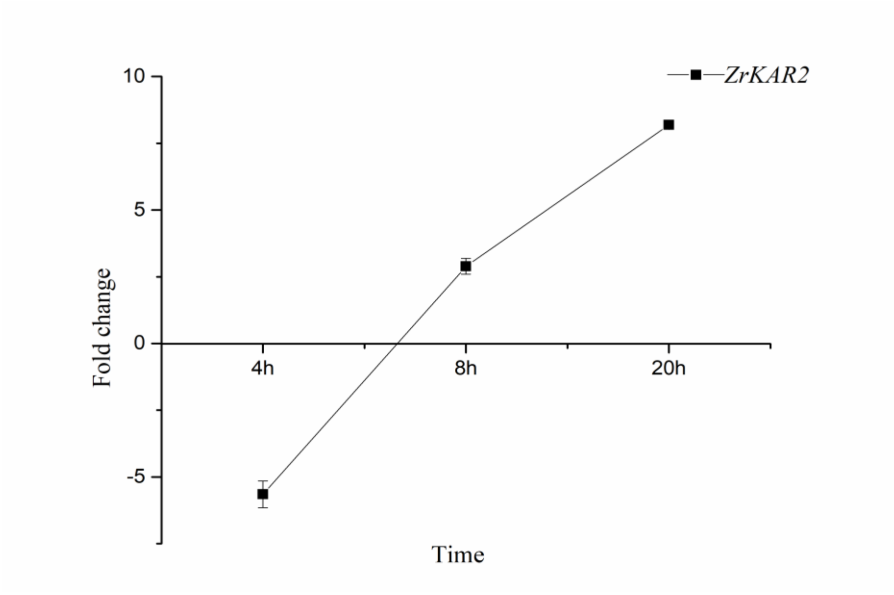

Figure S4 The RNA levels was performed by q-PCR for *ZrKAR2* gene in *Z. rouxii* cultivated at 60%w/v high concentrations.
